# Australian Podiatry Conference 2019: Adelaide, Australia. 22-24 May 2019

**DOI:** 10.1186/s13047-019-0352-z

**Published:** 2019-09-25

## Oral presentations

### O1 How population data informs the science of foot disorders and health

#### Marian Hannan

##### Marcus Institute for Aging Research, Hebrew SeniorLife and Harvard Medical School, Boston, MA, USA

This presentation will provide an epidemiological overview of the current population levels of foot pathologies, including current challenges and viewpoints. This session will also addressed the need for a data-driven approach as we consider common etiologies and causal pathways, for example, the links between obesity and pedal pathologies as well as special considerations regarding clinical trial designs for the foot and ankle research.

The past decade has brought many new insights to the epidemiology of foot and ankle disorders as well as insights into early pathology, and even possible prevention of impaired foot structure and function. Clinical cases and laboratory studies have provided information on treatment and insights into mechanisms. Yet, we still know relatively little of the population impact and informed prevention that may help people NOT become patients.

The objective of this presentation is to provide a population-based understanding of foot type, pathologies and function in the population from major studies in the United States. We will consider how populations inform science and medicine, how to obtain complex measurements from large groups outside the laboratory, and highlight major findings of population-based foot studies. A better understanding of these issues can help to inform the public as well as disseminate clinical and scientific information.

How do all of these data sources inform our understanding of foot biomechanics and translation of research? Epidemiology may serve as a bridge between our current knowledge base and how to grow this foundation to the next level of insights and interventions in the 21^st^ century. Such a focus will encourage the integration of our knowledge of biomechanics and movement with “Big Data” collections, taking our field to the next level.

### O2 The value of population data on foot problems: perspectives from Australia and the United Kingdom

#### Hylton Menz^2,1^

##### ^1^La Trobe Sport and Exercise Medicine Research Centre, La Trobe University , Melbourne, VIC, Australia; ^2^School of Allied Health, La Trobe University, Bundoora, VIC, Australia

Epidemiology can be defined as the study of how often diseases occur in different groups of people and the factors that may influence patterns of disease distribution in the community. By understanding the prevalence and risk factors for a particular disease, its burden can be quantified, and targets for treatment and prevention can be identified. Epidemiology is therefore a cornerstone of public health and provides valuable information for shaping health policy.

Although it has long been recognised that foot problems are highly prevalent, disabling and costly, foot problems have historically been ignored in population-based epidemiology studies. This lack of epidemiological data has prevented the podiatry profession from being able to demonstrate its value in relation to public health and has made it difficult for researchers to attract research funding. Fortunately, this has changed in recent years, with the initiation of several well-designed population-based studies incorporating detailed information on foot problems.

This presentation will provide an overview of five population-based studies that the presenter has had the privilege of being involved in over the past decade: (i) the North West Adelaide Health Study, (ii) the Australian Longitudinal Study of Women’s Health, (iii) the Geelong Osteoporosis Study, (iv) the Tasmanian Older Adult Cohort Study and (v) the Clinical Assessment Study of the Foot. Strengths and limitations of each of these studies will be discussed, along with a summary of their key findings. Finally, the presentation will discuss the recent establishment of a foot and ankle consortium to enhance research capacity and facilitate international collaboration using these resources.

### O3 Global update on physical activity – are we making progress in Australia and internationally?

#### Adrian Bauman

##### University of Sydney, Sydney University, NSW, Australia

Physical inactivity is a major contributor to the global burden of disease, both in high income earned in low middle income countries. Despite the advocacy efforts of NGOs, professional societies, and the Lancet series on physical activity in 2012 and 2016, much remains to be done to increase population levels of activity. This talk will review the policy framework and efforts to promote physical activity in Australia and internationally over the past three decades. Despite having many physical activity researchers, and having a national NCD plan, Australia has no physical activity plan or strategy to promote physical activity or reduced sitting time. Since 1989, rates of physical activity amongst Australian adults have remained unchanged or declined slightly, especially with respect to moderate to vigorous forms of structured physical activity and exercise. Rates of inactivity are also high amongst adolescents and children. The one area which may show some improvement is in population levels of walking, which is an accessible and feasible activity for almost all adults of all ages. Globally, there are also many challenges to resourcing physical activity strategies at the population level. These challenges posed an urgent public health crisis.

### O4 Athlete data and relevance for public health and physical activity across the lifespan

#### Michael K Drew^1,2,3^

##### ^1^Athlete Availability Program, Australian Institute of Sport, Canberra, ACT, Australia; ^2^Australian Centre for Research into Injury in Sport and its Prevention, Melbourne, VIC, Australia; ^3^Research Institute for Sport and Exercise , University of Canberra, Canberra, ACT, Australia

Olympic athletes provide an opportunity to understand the n^th^ degree of human potential. One of these potentials is the ability to withstand high workloads – both physical and mental. This lecture will outline the current activities undertaken by the Athlete Availability Program at the Australian Institute of Sport and detail the requirements to keep athletes healthy. A key focus of the talk will be how learnings can be translated to ‘everyday athletes’ and emerging areas that all clinicians should be aware of when managing injuries in active populations whether they are recreational or Olympians.

### O5 Training athletes to optimise performance

#### Jon Buckley

##### Alliance for Research in Exercise, Nutrition and Activity (ARENA), University of South Australia, Adelaide, SA, Australia

Athletes undergo training to induce adaptations that can improve performance. Too little training can result in suboptimal performance but, conversely, too much training with inadequate recovery can also lead to adverse performance effects. In order to optimise athletic performance training programs must achieve the correct balance between training load and recovery.

Technological advances have allowed for training loads to be quantified quite readily, but quantification of the load being performed does not provide any insight into how an athlete is responding to that load. A large number of biomarkers have been evaluated to determine how athletes are responding to different training loads, but currently there is insufficient evidence to support the use of any given biomarker.

Our team has been evaluating various parameters that can be assessed during submaximal exercise that correlate with changes in athletic performance following changes in training load. These parameters include heart rate acceleration during the transition from rest to exercise, and changes in movement and gait patterns. This presentation will provide insights into these novel methods for assessing how an athlete is responding to training, and how they can be used to inform changes in athletic training programs to optimise performance.

### O6 Efficacy of heel lifts in the treatment of mid-portion Achilles tendinopathy: a randomised trial

#### Chantel L Rabusin^1^, Hylton B Menz^1^, Jodie A McClelland^1^, Angela M Evans^1^, Karl B Landorf^1^, Peter Malliaris^2^, Sean I Docking^1^, Shannon E Munteanu^1^

##### ^1^La Trobe Sport and Exercise Medicine, La Trobe University, Melbourne, Victoria, Australia; ^2^Department of Physiotherapy, Monash University, Frankston, Victoria, Australia

###### **Correspondence:** Chantel L Rabusin

**Background**

Mid-portion Achilles tendinopathy (AT) is a common overuse musculoskeletal condition causing pain and disability. There are two commonly used conservative interventions. Calf muscle eccentric exercise has been found to be effective in decreasing pain and improving function in people with AT. Heel lifts reduce ankle joint dorsiflexion and Achilles tendon strain, however the efficacy of this intervention for AT has not been determined. The aim of this trial, therefore, was to compare the efficacy of heel lifts against calf muscle eccentric exercise for reducing pain and improving function in AT.

**Methods**

One hundred participants aged between 22 to 66 years (45.9 ± 9.4) with AT were randomised to receive either Clearly Adjustable^®^ 12 mm heel lifts (n = 50) or a calf muscle eccentric exercise program (n = 50). Outcome measures were obtained at baseline, 2, 6 and 12 weeks, the primary end-point. The primary outcome measure was the Victorian Institute of Sports Assessment-Achilles questionnaire (VISA-A). Secondary outcome measures included thickness and integrity of the Achilles tendon, global impression of change (pain and function), severity of pain (100 mm visual analogue scale) and calf muscle function (calf rise test). Data was analysed using the intention to treat principle. VISA-A and global impression of change in symptoms at 12 weeks are presented below.

**Results**

Both groups improved in pain and function. After 12 weeks, there was no significant difference in VISA-A scores between the two groups (adjusted mean difference at week 12 = 4.5 points, 95% CI -3.0 to 12.1, p=0.232). However, statistically significant differences between groups were found for patient global impression of change in symptoms in favour of heel lifts at 12 weeks (pain: RR 1.41, CI 1.02 to 1.95; function RR 1.55, CI 1.04 to 2.31).

**Discussion and clinical relevance**

There is no difference in efficacy between heel lifts and calf muscle eccentric exercise for AT. Heel lifts can be considered a simpler and alternative intervention to calf muscle eccentric exercise for AT.

### O7 Profiling footwear use, training habits, running related injuries, and injury management behaviours in recreational runners in the Australian community

#### Benjamin J Peterson^1^, Martin J Spink^1^, Fiona E Hawke^1^, Vivienne H Chuter^1^, Robin Callister^2^

##### ^1^School of health sciences, The University of Newcastle, Ourimbah, NSW, Australia; ^2^School of Biomedical Sciences and Pharmacy, The University of Newcastle, Callaghan, NSW, Australia

###### **Correspondence:** Benjamin J Peterson

**Background**

Running-related injuries (RRI) have become more prevalent as recreational running has gained popularity. The aetiology of RRI is considered to be multifactorial, and related to both intrinsic and extrinsic factors. This study describes the demographics, training habits, footwear selection, and injury patterns in recreational runners in the Australian community.

**Methods**

Recreational runners were recruited through community running events in New South Wales and completed self-report surveys, regarding footwear use, training habits, previous injuries, and demographic data. Descriptive statistics, correlations, and regression analyses were performed.

**Results**

Ninety-five runners (56 females) were recruited. Mean age was 47.2 + 13.0 years, weight 75.4 + 19.0 kg and height 169.3 + 9.2cm. Runners completed 2.87 + 1.39 weekly running sessions, covering 19.48 + 17.0 km per week, most commonly on concrete (58.9%; n=56) and bitumen (51.5%; n=49).

Footwear was prescribed by a podiatrist or footwear retailer for 20% (n=19) of runners, with 57.9% (n=55) using a neutral shoe and 25.3% (n=24) using motion-controlling footwear. Asics was the most commonly worn footwear brand (24.2%; n=23) and Asics Kayano the most commonly worn shoe overall (10.5%; n=10). The average age of current running shoes was 11.33 + 10.1 months. Increased weekly mileage was associated with a lower shoe age (r=0.324, p=0.002).

The rate of RRI within three months prior to recruitment was 50% (n=49). The most common injury sites were the ankles (20%; n=13), feet (16.9%; n=11), and knees (15.4%; n=10). Foot orthoses were worn by 20% (n=19) of runners and use was associated with previous injury (r=0.242, p=0.019). One third (n=15) of runners who sustained a RRI reported seeing a health professional, most commonly podiatrists (n=4) and physiotherapists (n=4). Those who saw a health professional were more likely to wear foot orthoses (r=0.441, p=0.002), and to have less time-loss from running, however this did not reach statistical significance (r=-0.272, p=0.061).

**Discussion and Clinical Relevance**

Injuries are common among Australian recreational runners. Injured runners frequently seek the advice of health professionals, and this behaviour may result in less time-loss due to injury, however further research with larger sample sizes is required to confirm this.

### O8 A systematic review into the diagnostic accuracy of clinical tests for detecting Posterior Tibial Tendon Dysfunction

#### Stephanie Lubcke, Ryan S Causby, Ian Fulton, Sandy Maranna, Steve Milanese

##### School of Health Sciences, University of South Australia, Adelaide, SA, Australia

###### **Correspondence:** Ryan S Causby

**Background**

Posterior Tibial Tendon Dysfunction (PTTD) is a progressive foot condition caused by overuse or degeneration of the Posterior Tibial Tendon. The condition is seen in orthopaedic, podiatry and physiotherapy clinics. Diagnosis is attained primarily through clinical tests and occasionally confirmed with MRI or ultrasound.

However, the evidence supporting the clinical tests is not clear. Thus, to determine the diagnostic accuracy of clinical tests for detecting PTTD in people with medial foot pain and associated symptoms we undertook a systematic review.

**Methods**

Searches of AMED, Medline, Embase, CINAHL, PubMed and Scopus were conducted. Two independent reviewers were involved in study selection and quality appraisal using Covidence and the QUADAS-2 tool, respectively.

**Results**

The initial database search yielded 813 articles. After title and abstract screening and duplicate removal, 47 studies remained. From these, 42 studies were excluded based on set criteria such as study population, study design, having a suitable index test and language; leaving five studies which had investigated the diagnostic accuracy of clinical tests for PTTD.

These studies comprised nine clinical tests, including: Unipedal standing balance test, Posterior Tibial Edema Sign, First Metatarsal Rise Sign, Single Leg Heel Raise (SLHR), Double Heel Raise, ‘Too many toes sign’ and Passive and Active muscle testing. The most common being the SLHR. Of the included studies, MRI was the reference standard in three studies, surgical exploration in one study and a control group was used in one study instead of a reference standard. Ultrasound was also utilised in two studies as an additional comparison reference standard.

However, all studies reviewed were at risk of bias and had concerns regarding their applicability.

**Discussion and Clinical Relevance**

This review finds that there is limited evidence of the diagnostic accuracy of clinical tests for PTTD. A discussion of the findings of the review and the identified clinical tests will be provided, with the aim of providing clinicians with the evidence underpinning tests commonly used in podiatry practice. Future research should be conducted into this area with a focus on high quality, well-powered studies investigating different clinical tests compared to a gold standard.

### O9 A cross-sectional study comparing clinical and psychosocial features in tibialis posterior tendinopathy with controls: preliminary findings

#### Megan H Ross, Michelle D Smith, Bill Vicenzino

##### The University of Queensland, St Lucia, QLD, Australia

###### **Correspondence:** Megan H Ross

**Background**

Tibialis posterior tendinopathy is associated with pain during activities that load the tibialis posterior tendon and limited participation in daily and physical activities. Little is known about factors such as quality of life and psychosocial features in tibialis posterior tendinopathy. The aim of this preliminary study was to investigate clinical and psychosocial characteristics of individuals with tibialis posterior tendinopathy.

**Methods**

We compared individuals with tibialis posterior tendinopathy (currently n = 19, age: 42±14; BMI: 27±8) and asymptomatic controls (n = 26, age: 43±17, BMI: 23±5). Diagnosis was made clinically based on medial foot/ankle pain plus one or more of: tenderness on palpation or swelling of the tibialis posterior tendon or pain/difficulty with resisted plantarflexion inversion or single leg heel raise. Clinical measures of foot posture, function, range of motion, strength at the foot and hip, and self-reported psychosocial measures were assessed. Preliminary between group differences were explored using independent t-tests. Standardised mean differences (SMDs) were calculated to evaluate effect sizes. Interpretation of SMDs was as follows: <0.6 small, 0.61-1.2 moderate, >1.2 large.

**Results**

Preliminary analyses suggest large effects (SMD > 1.2) for more pain, poorer self-reported function and quality of life, in addition to more pronated foot posture, poorer single leg heel raise endurance and greater time to complete stair descent/ascent in tibialis posterior tendinopathy compared to controls (p<0.01). Data collection is still ongoing and results will be updated at the conference.

**Discussion and Clinical Relevance**

Altered foot posture and poorer physical function appear to be accompanied by pain, stiffness, social limitations, and lower quality of life in tibialis posterior tendinopathy. A biopsychosocial approach should be considered for assessment and management of tibialis posterior tendinopathy.

### O10 The WHO Global Action Plan for Physical activity, taking the world to a more active 2030

#### Adrian Bauman

##### University of Sydney, Sydney University, NSW, Australia

In 2017, the World Health Organisation started the process of developing a global action plan for physical activity (GAPPA). This was a major step forward by WHO as part of its commitment to non-communicable disease prevention. This talk will describe the process of developing GAPPA, as it was somewhat different to usual WHO planning processes. Unlike the usual health-centric consultation, WHO used a broader and cross-sectoral base for the consultations around physical activity, including discussions with a broad range of health professionals, sport, education, urban planning and transport, all of whom have an important direct or indirect role in physical activity promotion. WHO has also set targets of a 10% relative reduction in physical inactivity by 2025, and a 15% reduction by 2030. The global action plan, launched in 2018, was comprised of four sections: [i] creating active societies and changing social norms to become more supportive of physical activity in everyday life; [ii] creating environments that are conducive to active living; [iii] supporting people through health sector advice and counselling to be more active, and including physical activity promotion in clinical consultations; and [iv] building an integrated system in which physical activity is easier in society, which involves collaboration across agencies and sectors, to create a more active world. This is a bold action plan by WHO and warrants as much support and advocacy to governments at every level as we can muster.

### O11 Avoid tripping over obesity

#### Gary Wittert

##### Freemasons Foundation Centre for Men's Health, University of Adelaide, Adelaide, SA, Australia

Excessive accumulation of lipid laden adipose tissue has adverse effects on health. These are due to the mechanical effects of excess body weight, metabolic/inflammatory consequences related to the anatomical distribution of the adipose tissue, concomitant co-morbid conditions, the effects of a range of health-related behaviours and psychosocial factors including stigma. Increased amounts, and more importantly function, of skeletal muscle abrogates the deleterious effects of adipose tissue.

Obesity has been reported to be associated with planus foot posture and when walking- pronated dynamic foot function and increased mid-foot plantar pressures, nonspecific foot pain and plantar heel pain. The amount and distribution of adipose tissue and presence of depression, but not lean mass or BMI have been shown to be independent mediators of obesity related foot pain

Use of podiatry services is significantly increased in people with moderate to severe obesity as compared to those people of healthy weight or mild obesity. Accordingly, podiatrists are well placed not just to manage end organ consequences of obesity and complications of obesity, but also to address the underlying cause and prevent further problems. This presentation will provide some suggestions for how that may be done.

### O12 Fat mass, but not fat-free mass, predicts increased foot pain with morbid obesity, independent of bariatric surgery

#### Tom P Walsh^1,2^, Stephen J Quinn^3^, Angela M Evans^4^, Alison Yaxley^1^, Jacob A Chisholm^5^, Lilian Kow^5^, E Michael Shanahan^1,5^

##### ^1^Flinders University, Bedford Park, South Australia, Australia; ^2^School of Clinical Sciences, Queensland University of Technology, Kelvin Grove, QLD, Australia; ^3^Swinburne University of Technology, Melbourne, Victoria, Australia; ^4^La Trobe University, Bundoora, Victoria, Australia; ^5^Flinders Medical Centre, Bedford Park, South Australia, Australia

###### **Correspondence:** Tom P Walsh

The full article version of this abstract has already been published and can be found at https://www.sciencedirect.com/science/article/abs/pii/S1550728918303290.

### O13 Bone marrow oedema is important: medical imaging findings in people with and without plantar heel pain

#### Karl B Landorf^1^, Hylton B Menz^1^, Michelle R Kaminski^1^, Gerard V Zammitt^2^, Tom Entwisle^3^, David Connell^3^, Shannon E Munteanu^1^

##### ^1^La Trobe University, VIC., Australia; ^2^Medical Foot Care, Altona North, Victoria, Australia; ^3^Imaging @ Olympic Park, Melbourne, Victoria, Australia

###### **Correspondence:** Karl B Landorf

**Background**

Medical imaging is frequently used to assist in the diagnosis of plantar heel pain (PHP). However, no studies investigating the association of imaging features with PHP have combined x-ray, ultrasound and MRI, nor have they used a control group matched for important characteristics. The aim of this study was to investigate the association of imaging findings with PHP.

**Methods**

In this cross-sectional study, 50 participants with PHP were compared to 25 participants without PHP (i.e. controls) matched for age, sex and body mass index (BMI). Imaging included x-ray, ultrasound and MRI.

**Results**

On x-ray, participants with PHP were 2.4x (95% CI 0.8, 6.7) more likely on the right and 3.7x (95% CI 1.3, 10.5) more likely on the left foot to have a plantar calcaneal spur. On ultrasound, plantar fascia thickness was 0.85 mm (95% CI 0.31, 1.40) thicker on the right and 0.74 mm (95% CI 0.23, 1.25) thicker on the left foot. Hypoechogenicity was 5.2x (95% CI 1.8, 14.7) more likely in the right and 3.0x (95% CI 1.1, 8.0) more likely in the left plantar fascia. On MRI, delamination was 5.0x (95% CI 1.7, 14.3) more likely in the right and 3.6x (95% CI 1.3, 10.0) more likely in the left plantar fascia. A tear of the plantar fascia was at least 9.3x (95% CI 1.2, 75.5) more likely in the right and at least 5.9x (95% CI 1.2, 28.2) more likely in the left foot. Bone marrow oedema of the calcaneus was 10.6x (95% CI 2.3, 49.9) more likely in the right and 5.3x (95% CI 1.4, 20.1) more likely in the left foot.

**Discussion and Clinical Relevance**

Our study is the first of its kind to compare participants with and without PHP when matched for age, sex, and significantly, BMI. Our findings highlight that PHP does not just involve the plantar fascia. While pathology of the plantar fascia is part of the clinical picture, increased odds of bone marrow oedema – an indication of bone stress – is an important finding from our study. Our findings may impact treatment of this condition.

### O14 The reliability of a two-probe ultrasound imaging procedure to measure strain in the Achilles tendon

#### Prue Molyneux, Richard Ellis, Matthew Carroll

##### Auckland University of Technology, Northcote, AUCKLAND, New Zealand

###### **Correspondence:** Prue Molyneux

**Background**

Alteration in the strain properties of the Achilles tendon may lead to adaptations such as pathological stiffening. Stiffer tendons have less adaptive ability, which may predispose to the development of tendinopathy. There is a current lack of methods to measure in-vivo tissue strain. A two-probe ultrasound procedure has been proposed to reduce measurement error associated with a one-probe procedure. However, reliability of the two-probe procedure has not been established. The study aimed to determine intra-rater, inter-session and inter-rater reliability of a two-probe ultrasound procedure to measure Achilles tendon strain.

**Methods**

Twentynine healthy participants (19 females, 10 males mean age 33.6 years) were included. Achilles tendon images were acquired with a two-probe ultrasound procedure as the ankle moved through a standardised range of motion (20° plantarflexion to 10° dorsiflexion). Both probes were positioned longitudinally, one probe over the musculotendinous junction and the second over the calcaneal insertion of the Achilles tendon. Repeat measurements were taken at a second session at the initial study visit and four weeks later. Strain measures were calculated from the pre-captured images using Motion Analysis 2014v1 software by two independent raters. Intra-rater, inter-session and inter-rater reliability were calculated using intraclass correlation coefficients (ICC). In addition, 95% confidence intervals for the ICC and the standard error of measurement (SEM) were also calculated.

**Results**

Excellent intra-rater within-session reliability (ICC = 0.84, *P* < 0.001), poor inter-session reliability (ICC = 0.18, p = 0.397) and excellent inter-rater reliability (ICC = 0.88, p = 0.003) was demonstrated for the measurement of Achilles tendon strain using the two-probe procedure.

**Discussion and Clinical Relevance**

The two-probe procedure to measure Achilles tendon strain is reliable for repeated measurements within the same day. However, measurement error increased when strain was measured on different days, which may be due to a combination of examiner error and participant factors. Measurement of Achilles tendon strain offers an additional tool for the evaluation of the tendon’s mechanical characteristics. The ability to reliably quantify strain may allow clinicians to identify those at risk of Achilles tendinopathy and to formulate more effective management plans.

### O15 Foot orthoses or corticosteroid injection for plantar heel pain: which is more effective and who is more likely to respond?

#### Glen A Whittaker^1^, Shannon E Munteanu^1^, Hylton B Menz^1^, James M Gerrard^1^, Ayman Elzarka^2^, Karl B Landorf^1^

##### ^1^La Trobe University, Bundoora, Victoria, Australia; ^2^Southern Cross Medical Imaging, Bundoora, Victoria, Australia

###### **Correspondence:** Glen A Whittaker

**Background**

Plantar heel pain is a common foot complaint that causes significant disability and poor health-related quality of life. Foot orthoses and corticosteroid injection are effective treatments for plantar heel pain, however it is unclear if one is more effective than the other. It is also unclear whether there are characteristics that can predict who is more likely to respond to each treatment. The aim of this research was to: (i) evaluate the effectiveness of foot orthoses and corticosteroid injection, and (ii) investigate if any parameters can predict who is more likely to respond to each treatment.

**Methods**

A parallel-group, assessor-blinded randomised trial (the SOOTHE trial) was conducted. Participants were randomly allocated to receive either prefabricated, arch-contouring foot orthoses or a single ultrasound-guided corticosteroid injection. The primary outcome measure was the foot pain subscale of the Foot Health Status Questionnaire at 4 and 12 weeks. To define responders, response to treatment was achieved by dichotomising each participant’s overall improvement measured on a 15-point Likert scale.

**Results**

A total of 103 participants aged 21 to 72 years (63 female) with plantar heel pain were recruited from the community and received an intervention. For the primary outcome of foot pain, corticosteroid injection was more effective at week 4 (adjusted mean difference 8.2 points; 95% CI 0.6, 15.8). However, foot orthoses were more effective at week 12 (adjusted mean difference 8.5 points; 95% CI 0.2, 16.8). Although these findings were statistically significant, they did not meet the previously calculated minimal important difference value of 12.5 points. The analysis of parameters that predict response is underway and will be complete prior to the conference.

**Discussion and clinical relevance**

Based on the findings of this trial, health professionals can advise patients with plantar heel pain that a single ultrasound-guided corticosteroid injection is effective, however the effect is relatively short-lived. However, this may be extended with appropriately contoured foot orthoses, which are more effective in the longer-term. Parameters that predict response will be discussed when the data analysis is complete.

### O16 Fifty active years after 50®? The challenges of exercise and activity with joint pain

#### Anne-Maree Keenan^1,2^

##### ^1^Faculty of Medicine and Health, University of Leeds, Leeds, United Kingdom; ^2^NIHR Leeds Biomedical Research Centre , Leeds Teaching Hospitals Trust, Leeds, United Kingdom

Musculoskeletal conditions (MSK) conditions are the single most common cause of chronic disability and one of the most expensive to treat. The global burden of MSK diseases has increased by 25 percent over the past decade. The musculoskeletal conditions that have the largest economic and personal burden are immune mediated inflammatory diseases (IMIDs) and osteoarthritis (OA). IMIDs affect over 5% of the population and result in joint damage, physical disability and reduced life expectancy. While not as aggressive, OA presents a wider challenge: worldwide estimates of OA indicate that one in ten men and one in five women aged over 60 have symptomatic OA and treatment options remain poor. The prevalence of OA will increase with changing ageing and obesity rates, with an expected increase in joint replacement surgery in younger people who will subsequently be living longer with their replacements, requiring thought to be placed on how we can sustain 50 active years after 50®.

While research has established that exercise is key in reducing symptoms, long-term damage and the impact of multi-morbidities, two main issues are evident in people with joint pain. First, in those with IMIDs, health professionals have been responsible for providing confusing advice aimed avoiding exercising and activity. While this has been driven by the premise of avoiding long term harm, it has resulted in both physical and psychosocial impact. Second, while people may understand the importance of exercise for long term benefit for preventing and reducing symptoms in OA, promoting exercise adherence and activity adoption remains the key clinical challenge.

This presentation will provide an overview of the benefits of exercise, strategies which are aimed at promoting accurate information sharing, exercise adherence and activity engagement for those with joint pain.

### O17 Midfoot pain and osteoarthritis: finding solutions to keep people on their feet

#### John Arnold

##### University of South Australia, Adelaide, SA, Australia

Midfoot osteoarthritis (OA) is a common cause of midfoot pain, affecting 1 in 8 people aged over 50 years. Often, midfoot OA is sufficiently painful to stop people walking. Once this occurs, people descend on a spiral of disadvantage characterised not just by painful feet, but all the established negative sequelae of inactivity. Clinically, OA in the midfoot can be challenging to treat with variable effectiveness of conservative interventions. Our ability to design effective treatments for midfoot OA has been hampered by a limited understanding of the modifiable determinants of disease initiation and progression. This is underpinned by two issues. First, early structural changes in midfoot OA remain undefined and may require MRI to measure accurately and precisely. Second, while joint loading is a key factor in the development and progression of OA at other weight-bearing joints, its role in midfoot OA is not well understood. This presentation will focus on recent research being conducted to unravel the mechanisms by which midfoot OA may develop and progress. It will also cover clinical factors related to symptoms and functional impairment that are future targets for intervention in clinical practice. Ultimately, improving the treatment of midfoot OA presents an opportunity to reduce the negative downstream effects of pain and foot-related disability on physical inactivity and general health.

### O18 Moving more and sitting less in the workplace

#### Tracy Kolbe-Alexander

##### University of Southern Queensland, Ipswich, QLD, Australia

The prevalence of non-communicable diseases (NCD’s) such as diabetes and coronary artery disease is increasing both globally and in Australia. The prevalence of diabetes in Australia has increased by approximately 200% in the past 20 years, and cardiovascular disease remains the leading cause of death in Australians. Physical inactivity is one of the main risk factors for non-communicable diseases like diabetes and cardiovascular disease. If all inactive Australians became physically active, 10% of deaths (all-cause mortality) could be prevented. Similarly, deaths due to coronary heart disease and Type II diabetes would decrease by 6% and 8%, respectively, if inactive Australians became physically active.

Prevention of life style and work related diseases include implementation of physical activity (PA) and the worksite has been identified as an appropriate setting for health promotion. To date, several initiatives have been taken by employers to promote a healthy lifestyle including the promotion of physical activity. More recently, workplaces have implemented various initiatives to reduce sitting and promote moving in the workplace.

This talk will include the recently updated physical activity guidelines. Evidence-based practice of various workplace and community based health promotion programs that aim to promote physical activity will be discussed. In addition, practical guidelines and recommendations to encourage working adults to move more and sit less will be presented.

### O19 Efficacy of shoe-stiffening inserts for the treatment of first metatarsophalangeal joint osteoarthritis: preliminary findings from the SIMPLE randomised controlled trial

#### Shannon E Munteanu^1,2^, Karl B Landorf^1,2^, Jodie A McClelland^2,3^, Edward Roddy^4^, Flavia M Cicuttini^5^, Alan Shiell^6^, Maria Auhl^1,2^, Jamie J Allan^1,2^, Andrew K Buldt^1,2^, Hylton B Menz^1,2^

##### ^1^Discipline of Podiatry, School of Allied Health, La Trobe University, Melbourne, Victoria, Australia; ^2^La Trobe Sport and Exercise Medicine Research Centre, School of Allied Health, La Trobe University, Melbourne, Victoria, Australia; ^3^Discipline of Physiotherapy, School of Allied Health, La Trobe University, Melbourne, Victoria, Australia; ^4^Arthritis Research UK Primary Care Centre, Research Institute for Primary Care and Health Sciences, Keele University, Staffordshire, United Kingdom; ^5^Department of Epidemiology and Preventive Medicine, Monash University, Alfred Hospital, Melbourne, Victoria, Australia; ^6^School of Psychology and Public Health, La Trobe University, Melbourne, Victoria, Australia

###### **Correspondence:** Shannon E Munteanu

**Background**

The purpose of this trial was to assess the efficacy of shoe-stiffening inserts for reducing pain associated with first metatarsophalangeal joint (MTPJ) osteoarthritis (OA).

**Methods**

One hundred participants (45 men 55 women, mean age 57.5, SD 10.3 years) with first MTPJ OA were randomised to receive either: (i) full-length carbon fibre shoe-stiffening inserts plus rehabilitation therapy (intervention group) or (ii) sham shoe inserts plus rehabilitation therapy (control group). The primary outcome measure was the foot pain domain of the Foot Health Status Questionnaire (FHSQ) assessed at 12 weeks. Secondary outcome measures included the function domain of the FHSQ, severity of first MTPJ pain, general health status, use of rescue medication and co-interventions, adverse events, physical activity levels and global change in symptoms (with ‘moderately better’ or above being considered a successful outcome). FHSQ and global change in symptoms at 12 weeks are presented here. Multiple imputation was used to replace missing data. FHSQ pain scores were analysed using analysis of covariance with the intervention group and baseline scores entered as independent variables, and global change in symptoms was analysed using absolute and relative benefit, and number needed to treat.

**Results**

Data were available for 91 participants at week 4 and 85 participants at week 12. FHSQ pain scores improved in both groups. There was a statistically significant difference in FHSQ pain scores between the groups at 12 weeks (adjusted mean difference 7.4 points, 95% confidence interval [95% CI] 1.3 to 13.5, p=0.018) in favour of intervention group. Participants in the intervention group were also more likely to report that their pain was at least moderately better (successful treatment) at 12 weeks compared to the control group (61 versus 34%; absolute benefit increase 27% [95% CI 6 to 45], relative benefit increase 79% [95% CI 11 to 189]). The number needed to treat was 4 (95% CI 2 to 18).

**Discussion and Clinical Relevance**

Carbon fibre shoe stiffening inserts are effective at reducing pain in people with first MTPJ OA.

### O20 A multi-faceted podiatry intervention compared to usual general practitioner care for first metatarsophalangeal joint osteoarthritis: a randomised controlled feasibility study

#### Kade L Paterson^1^, Rana S Hinman^1^, Ben r Metcalf^1^, Penny K Campbell^1^, Hylton B Menz^2^, David J Hunter^3^, Kim L Bennell^1^

##### ^1^The University Of Melbourne, Parkville, VIC, Australia; ^2^School of Allied Health, La Trobe University , Melbourne; ^3^Northern Clinical School, Kolling Institute of Medical Research, Institute of Bone and Joint Research, University of Sydney, Sydney

###### **Correspondence:** Kade L Paterson

**Background**

Osteoarthritis (OA) of the first metatarsophalangeal (MTP) joint is highly debilitating with limited evidence for treatment options. This study aimed to determine the feasibility of a randomised controlled trial comparing a multi-faceted podiatry intervention to usual general practitioner (GP) care for people with first MTP joint OA.

**Methods**

People aged >45 years with symptomatic radiographic first MTP joint OA were recruited from the community using advertisements and our existing networks. Participants in the intervention arm had 3-5 visits with a podiatrist and received prefabricated foot orthoses (>6hrs wear/day), home exercise and manual therapy (twice/day), and advice concerning footwear, weight loss, physical activity, and analgesia. Participants in the usual care arm had 1-2 visits with a GP and received medication advice/prescription, and the same advice as the intervention group. Primary outcomes were measures of feasibility assessed at 3-months (recruitment/retention rates, exercise sessions/week on an 11-point numerical rating scale (NRS), and orthoses wear hours/day). Secondary outcomes included walking pain (11-point NRS), function (foot health status questionnaire; FHSQ), global rating of change (7-point Likert Scale), adherence (11-point NRS), and adverse events.

**Results**

Thirty people from 236 screened (12.7%) were included. All except one attended the required clinical visits, and 26 completed final outcomes (14 in podiatry group, 12 in GP group). In the podiatry group, adherence was good with exercise (mean 9.3 sessions/week, 7.1/10 on NRS) and orthoses wear (6.2 hours/day, 7.0/10 on NRS). Adherence to medication use in the GP group was lower (5.3/10 on NRS). There were three reported minor adverse events in the podiatry group that all resolved. Both groups reported improved pain (mean change in podiatry group: -2.2/10, GP group: -2.8/10 on NRS) and function (podiatry: 18.3/100, GP: 13.6/100 on FHSQ) above minimum clinically important differences. Seven people (50%) in the podiatry group and 4 (33%) in the GP group rated themselves as “moderately better” or “much better”.

**Conclusion**

A clinical trial comparing a multi-faceted podiatry intervention to usual GP care for people with first MTP joint OA is feasible and safe, and both treatments improve symptoms. Results will be used to help power a larger clinical trial.

### O21 Structural characteristics associated with radiographical severity of 1^st^ metatarsophalangeal joint osteoarthritis

#### Andrew K Buldt^1,2^, Shannon E Munteanu^1,2^, Hylton B Menz^1,2^

##### ^1^Discipline of Podiatry, School of Allied Health, La Trobe University, Bundoora, Victoria, Australia; ^2^La Trobe Sport and Exercise Medicine Research Centre, School of Allied Health, La Trobe University, Bundoora, Victoria, Australia

###### **Correspondence:** Andrew K Buldt

**Background**

Osteoarthritis (OA) of the 1^st^ metatarsophalangeal joint (MTPJ) is a common and disabling condition. The aims of this study were to determine if differences exist in the foot structure of people with and without radiographically defined 1^st^ MTPJ OA, and whether structural differences are associated with radiographic severity of 1^st^ MTPJ OA.

**Methods**

Weight-bearing dorso-plantar and lateral radiographs were obtained for the symptomatic foot of 137 participants (60 men, 77 women, aged 22 to 85, mean 58.1 +/- 11.4 years) with clinically diagnosed 1^st^ MTPJ OA. Radiographic 1^st^ MTPJ OA cases were identified and participants were graded into four severity categories using a validated atlas. The following radiographic measurements were performed: hallux abductus angle, hallux abductus interphalangeus angle, intermetatarsal angle, metatarsal protrusion distance, metatarsus adductus angle, first metatarsal declination angle, lateral intermetatarsal angle, calcaneal inclination angle, 1^st^ metatarsal length and width, hallux proximal phalanx length and width and hallux distal phalanx length. Structural differences were compared using univariate general linear models, adjusting for age, sex and body mass index.

**Results**

One hundred and four participants were categorised as having radiographic 1^st^ MTPJ OA. The OA case group displayed a greater hallux abductus interphalageus angle compared to the no OA case group. Participants were further categorised into no OA (n=6), mild OA (n=27), moderate OA (n=49) or severe OA (n=55) categories. Participants of the mild, moderate and severe OA categories displayed a longer 1^st^ metatarsal compared to the no OA category. The moderate and severe OA categories displayed a greater hallux interphalangeus angle compared to the mild category OA.

**Discussion and clinical relevance**

Overall, there were few structural differences between OA cases and non-OA cases and between OA severity categories, although there was some evidence to suggest greater 1^st^ metatarsal length in more severe cases of 1^st^ MTPJ OA. The greater abduction of the hallux distal phalanx in OA cases and greater severity OA categories suggest a structural adaptation secondary to biomechanical factors. Further research is required to determine whether measures of dynamic foot function are associated with the presence and/or severity of 1^st^ MTPJ OA.

### O22 Experience of finding footwear and factors contributing to footwear choice in people with gout

#### Angela Brenton-Rule^1^, Nicola Dalbeth^3,2^, N Lawrence Edwards^4^, Keith Rome^1^

##### ^1^Department of Podiatry, Auckland University of Technology, Auckland, North Island, New Zealand; ^2^Department of Rheumatology, Auckland District Health Board, Auckland, New Zealand; ^3^Faculty of Medicine and Health Science, University of Auckland, Auckland, North Island, New Zealand; ^4^Department of Medicine, University of Florida, Gainesville, Florida, USA

###### **Correspondence:** Angela Brenton-Rule

The full article version of this abstract has already been published and can be found at https://jfootankleres.biomedcentral.com/articles/10.1186/s13047-018-0313-y.

### O23 Can ultrasound measures of intrinsic foot muscles and plantar soft tissues predict future diabetes-related foot disease? A systematic review

#### Troy Morrison^1,2^, Sara Jones^3,1^, Ryan S Causby^1,2^, Kerry Thoirs^1,2^

##### ^1^School of Health Sciences, University of South Australia, Adelaide, South Australia, Australia; ^2^International Centre for Allied Health Evidence , University of South Australia, Adelaide, South Australia, Australia; ^3^Department of Rural Health, University of South Australia, Adelaide, South Australia, Australia

###### **Correspondence:** Troy Morrison

The full article version of this abstract has already been published and can be found at https://journals.plos.org/plosone/article?id=10.1371%2Fjournal.pone.0199055.

### O24 The effectiveness of non-surgical interventions for common plantar digital compressive neuropathy (Morton's neuroma) a systematic review and meta-analysis

#### Barry G Matthews^1^, Sheree E Hurn^1,2^, Michael P Harding^3^, Rachel A Henry^4^, Robert S Ware^5^

##### ^1^School of Clinical Sciences, Queensland University of Technology, Brisbane, Queensland, Australia; ^2^Institute of Health and Biomedical Innovation, Queensland University of Technology, Brisbane, Queensland, Australia; ^3^School of Health Sciences, University of South Australia, Adelaide, South Australia, Australia; ^4^Rachel Henry Podiatry, Brisbane, Queensland, Australia; ^5^Menzies Health Institute Queensland, Griffith University, Brisbane, Queensland, Australia

###### **Correspondence:** Barry G Matthews

The full article version of this abstract has already been published and can be found at https://jfootankleres.biomedcentral.com/articles/10.1186/s13047-019-0320-7.

### O25 The child in the modern environment

#### Jennifer Couper

##### Womens and Childrens Hospital, Adelaide, SOUTH AUSTRALIA, Australia

Over the last 30 years non-communicable disorders have been on the rise in children: overweight/obesity, diabetes, inflammatory bowel disease, coeliac disease, allergy and neurodevelopmental disorders including autism spectrum disorder and attention deficit hyperactivity disorder. Potential common drivers from pregnancy and early life in the environment will be presented, using type 1 diabetes as the prototype to illustrate the effect of the modern environment on the at-risk child.

### O26 Exploring health professionals understanding of evidence and use of different treatment strategies to manage idiopathic toe walking

#### Cylie Williams^1^, Kelly Gray^2^, Nina Davies^3^, Marybeth Barkocy^4^, Michael Fahey^5^, Jane Simmonds^6^, Pasquale Accardo^7^, Deborah Eastwood^8^, Verity Pacey^2^

##### ^1^Monash University, Frankston, VIC, Australia; ^2^Macquarie University, Macquarie Park; ^3^Staffordshire University, Stoke on Trent; ^4^University of New Mexico, Albuquerque; ^5^Monash Children's Hospital, Clayton; ^6^Great Ormond Street Institute of Child Health, London; ^7^Virginia Commonwealth University School of Medicine, Richmond; ^8^Great Ormond St Hospital and University College, London

###### **Correspondence:** Cylie Williams

**Background**

Idiopathic toe walking (ITW) is an exclusionary diagnosis^1^ and varies in severity, from those children with full range of ankle motion^2^, to those with associated ankle equinus^2^. Many clinicians are faced with challenges in understanding available evidence-based treatment options in the absence of an evidence based treatment pathway. The primary aim of this research was to understand the agreement between health professionals’ knowledge of evidence for common treatment strategies for ITW and if the health professional's support these strategies being used in clinical practice.

**Methods:**

This was an international cross-sectional online survey between July 2017 and March 2018. This survey was open to registered health professionals who treat children with ITW. This survey was advertised through professional associations, social media and special interest groups. The survey had two components: 1) General demographic variables and variables relating to knowledge of evidence about ITW treatments and 2) Their support for common treatment strategies. Additional data on treatments, referrals, and preference were collected. The Kappa statistic was used to describe the intra-rater agreement between evidence knowledge and support. Regression analyses were used to understand strategy use preference of the 10 most commonly preferred treatments.

**Results:**

There were 908 international survey responses primarily from medical doctors (n=24), orthotists (n=80), physiotherapists (n=589) and podiatrists (n=149). Kappa agreement for paired correct responses determined a fair agreement for *evidence support knowledge* for four strategies including *Watch and Wait* (Kappa=0.24), *Stretching* (Kappa=0.30), *Sensory Integration Strategies* (Kappa=0.40) and *Motor Control Strategies* (Kappa =0.24) and moderate agreement for thirteen others. No strategies had greater than moderate agreement between knowledge of evidence and the support for the strategy being used. Profession, country of practice, average number of children treated per week, and not correctly identifying the evidence factored into many of the most commonly used strategies for ITW (p<0.05).

**Discussion and Clinical Relevance**

The results from this study confirm a large variety of interventions are utilised for the management of ITW around the world. Furthermore, there remains a disconnect between health professional's understanding of the evidence of common treatment strategies of ITW and a consensus for the treatment of this condition.

1. Williams CM, Tinley P, Curtin M: The Toe Walking Tool: a novel method for assessing idiopathic toe walking children. Gait & Posture 2010, 32:508—511. 2. Davies K, Black A, Hunt M, Holsti L: Long-term gait outcomes following conservative management of idiopathic toe walking. Gait & Posture 2018, 62:214-219.

### O27 Developmental coordination disorder in children and the interface with the podiatry profession

#### Mitchell Smith^1^, Helen Banwell^1^, Cylie Williams^2^, Emily Ward^1^

##### ^1^University of South Australia, Adelaide, SA, Australia; ^2^Monash University, Melbourne, VIC, Australia

###### **Correspondence:** Mitchell Smith

**Background:**

Developmental coordination disorder (DCD) is a common condition in children affecting motor coordination with resulting impacts on academic performance and activities of daily living. Literature surrounding intervention has focused mostly on physical and occupational therapies, however it is known that children with DCD are seen clinically by podiatrists due to abnormalities in gait and lower limb functioning. This presentation combines current clinical knowledge and practices of Australian podiatrists and children with DCD and the outcomes of a systematic review of differences in gait between children with and without DCD.

**Methods:**

A single-round survey, developed using SurveyMonkey®, was completed by a sample of Australian podiatrists through either online or paper means. Participants were asked about familiarity with DCD and depending on their response, were directed via skip logic to questions on presentation, assessment and management of DCD. Participants were also asked about willingness to receive further education on DCD. Descriptive statistics were used to present the data. A concurrent systematic review following PRISMA guidelines was conducted.

**Results:**

365 Australian podiatrists completed the survey. 30% reported familiarity with DCD, while 67% reported familiarity with alternate and outdated terminology. Those familiar showed good knowledge of signs and symptoms associated with DCD. Both familiar and unfamiliar participants favoured referral to other health professionals over completing assessments. Common podiatric management strategies such as footwear advice, orthoses, and strength training were the most frequently chosen by both groups, despite footwear and orthoses having no current evidence base for DCD. Participants were willing to receive education on DCD through a range of both online and in-person mediums. Through the systematic review, children with DCD were found to have reduced endurance and cardiorespiratory fitness than their typically developing peers.

**Discussion and Clinical Relevance:**

Lower endurance and fitness levels may contribute to the reduced participation of children with DCD. A majority of Australian podiatrists were unfamiliar with DCD, despite its prevalence and symptomology which falls within the podiatric scope. However, participants overwhelmingly showed willingness to receive education on DCD. Further research is required to establish the role of podiatrists in the assessment and management of children with DCD.

### O28 Comparison of hard soled shoes versus soft soled shoes in young children: Spatiotemporal measures of gait

#### Simone Cranage^1^, Luke Perraton^2^, Kelly-Ann Bowles^3^, Cylie Williams^4^

##### ^1^Podiatry, Peninsula Health, Frankston, VIC, Australia; ^2^Physiotherapy, Monash University, Melbourne, VIC, Australia; ^3^Director of Research, Monash University, Melbourne, VIC, Australia; ^4^Adjunct Research Lead, University of South Australia, Adelaide, SA, Australia

###### **Correspondence:** Simone Cranage

**Background**

There is little evidence to guide recommendations of footwear features, for young children, in particular the sole hardness. The aim of this study was to investigate the difference in spatiotemporal measures of gait in young children during walking and running in different types of soft and hard soled footwear

**Methods**

Demographic and lower limb anthropometric data was collected from typically developing children. Participants walked and ran along a GAITrite mat at a self-selected speed with the condition order randomized. Duplicate footwear was tested in a boot, runner and sandal with two different sole hardness; an exisiting industry standard (Shore A) and a comparative sole being 20% outside of tolerance range (Shore B). Spatiotemporal gait measures were extracted from the GAITrite. Linear regression clustered by participant was used to understand the different gait variables.

**Results**

There were 47 typically developing children aged 2-4 years recruited. Soft-soled sandals increased stride length compared to hard soled footwear (Coef=2.07, CI95%=-4.01 to -0.08, p=0.04) during walking only. There were no differences between walking or running in soft or hard soled sandals, boots or runners. There was a small increase in tripping in soft-soled sandals during walking only.

**Discussion and Clinical Relevance**

Current perception is that sole hardness is an important feature in young children’s shoes. These findings infer that sole hardness has a limited effect on the spatiotemporal measures of young children’s gait in walking and running. Parents seeking advice from health professionals about footwear and be informed that this shoe feature has limited impact on walking and running in younger children which can therefore guide both clinician and industry recommendations.

### O29 International normative data for paediatric foot posture – from over 3000 cases

#### Angela M Evans^1^, Gabriel Gijon- Nogueron^2^, Alfonso Martinez-Nova^3^, Pilar Alfageme-Garcia^3^, Jesus Montes-Alguacil^2^

##### ^1^Discipline of Podiatry, School of Allied Health, College of Science, Health and Engineering, La Trobe University, Melbourne, Victoria, Australia; ^2^Nursing and Podiatry, University of Malaga, Malaga, ES, Spain; ^3^Nursing and Podiatry, University of Extremadura, Extremadura, ES, Spain

###### **Correspondence:** Angela M Evans

**Background**

There is ongoing confusion about paediatric foot posture, and a lack of clearly defined values results in overdiagnosis of ‘flatfoot’, and frequently unnecessary treatment. The main objectives of our collaboration were to: enlarge reference data for foot posture across childhood, and to clarify the influence of basic anthropometry (BMI) on paediatric foot posture. It has long been cited that heavier children have flatter feet, yet also refuted. This study both doubles the previous normative data for paediatric foot posture, and extends the age range.

**Methods**

Amalgamation of datasets from cross-sectional studies in Spain, UK, and Australia was undertaken.

The final dataset comprised 3217 healthy children, aged from three to 15 years. Foot posture was described by means and z-score of the foot posture index (FPI). Height and weight of each participating child was used to calculate the body mass index (BMI), and percentiles were used to categorise BMI.

**Results**

A pronated foot posture (FPI ≥ +6) was found in 960 (29.8%), and a normal foot posture (FPI 0 to +6) in 1776 (55.2%) of children. The less common foot postures, highly pronated (FPI ≥ +10 ) were found in 127 (3.9%) children, and supinated foot (FPI < 0) in 354 (11.0%) children.

Approximately 20% of children were overweight/obese, but correlation between BMI and FPI was weak and inverse (r = -0.066, p< 0.01), further refuting the relationship between increased body mass and ‘flat’ or pronated feet.

**Discussion and Clinical Relevance**

This study confirms that the ‘flat’ or pronated foot is common in childhood, with FPI score of +4(3) the average finding across all ages. A wide normal range of foot posture across childhood is confirmed, with 68% of children having FPI range +1 to +7.

Clinicians should be aware that both highly pronated feet (FPI > +10), and supinated foot posture (FPI < 0) is unusual, and should prompt both differential diagnoses, and gait evaluation.

A foot posture versus age ‘ready reckoner’ has been produced for clinical use and parent education.

### O30 The foot-health and mortality of adult patients with diabetes in Northern Tasmania: findings from an epidemiological study with two-year follow-up

#### Byron Perrin^1^, Derek Condon^2^, Penny Allen^3^, Andrew Chappell^2^, Claire Massey^4^, Marcus Gardner^5^, Bronwyn Phillips^6^, Isabelle Skinner^7^, Timothy Skinner^8^

##### ^1^La Trobe University, Bendigo, VICTORIA, Australia; ^2^Tasmanian Health Service- North-West, Devonport, Tasmania, Australia; ^3^Rural Clinical School, University of Tasmania, Launceston, Tasmania, Australia; ^4^Tasmanian Health Service- North, Launceston, Tasmania, Australia; ^5^Bendigo Health, Bendigo, Victoria, Australia; ^6^Murray Primary Health Network, Bendigo, Victoria, Australia; ^7^International Council of Nurses, Geneva, Switzerland; ^8^Department of Psychology, University of Copenhagen, Copenhagen, Denmark

###### **Correspondence:** Derek Condon

**Background**

There is limited epidemiological research that reports on the foot-health of people with diabetes within Australian regional settings. The objective of this two-year follow-up analysis was to explore incident diabetes-related foot morbidity and mortality in people residing in Northern Tasmania.

**Methods**

Adults with diabetes were recruited from predominately community-based, publicly-funded podiatric services in regional Tasmania. The primary variable of interest was the incidence of foot ulceration, lower limb amputation and death. Other variables of interest were age, sex, rurality, socio-economic disadvantage, diabetes type and duration, knowledge of diabetes and smoking status. The main outcome was incidence of foot morbidity (foot ulceration, lower limb amputation or death) per 100 person-years. A survival analysis was conducted to determine median time to each morbidity outcome.

**Results**

There were 445 Tasmanian patients (264 males and 181 females) who completed baseline assessments. Mean age at baseline was 65 (SD 12.9, range 19-97). Sixty-two (13.9%) participants had type I diabetes and 383 (86.1%) had type II. Three hundred and fifty-three (79.3%) participants had at least one follow-up visit, with 285 (64.0%) participants still being followed-up at 12 months, 248 (55.7%) at 18 months and 203 (45.6%) at 24 months. Median number of follow-up visits = 10 (IQR 4, 22, range 1, 98). There were 57 deaths (12.8%). There were 157 (35.3%) new ulcers during the study period and 24 (5.4%) new amputations. Risk factors for worsening foot morbidity over time and the results of the survival analysis will also be presented.

**Conclusions**

Public podiatric services in regional Tasmania are managing patients at significant risk of serious diabetes-related foot morbidity. The two-year incidence of ulceration and amputation is high, and the proportion of participants who died after two years is an important finding in the Australian context. Patients presenting to these regional public podiatry services require multi-disciplinary health care in accordance with national and international guidelines. There is a potential disparity between current funding models for these services and the level of diabetes-related foot morbidity the services are managing.

### O31 Looking on the bright side of a diabetes diagnosis

#### Anna Horn, Luke Donnan, Emma Baker, Caroline Robinson

##### Charles Sturt University, Thurgoona, NEW SOUTH WALES, Australia

###### **Correspondence:** Anna Horn

**Background**

One in four adults over the age of 25 years are living with diabetes or what is known as pre-diabetes, and in 2016 diabetes was the seventh leading cause of death in Australia. It is therefore, no surprise that publically accessible diabetes resources commonly highlight the potential negatives associated with a diagnosis of diabetes, such as neurological and vascular complications, amputation, and higher rates of depression. What is less commonly highlighted is that half of people diagnosed with diabetes report coping well, and 72% are rarely restricted in their daily activities.

**Methods**

Random sampling was used to recruit 50 participants (31 female; 19 male; 71.78±9.64 years) to a foot health promotion event at the Charles Sturt University Community Engagement and Wellness Centre. Student practitioners completed basic neurovascular assessments to ascertain each participant’s arterial, venous and neurological status. Participants also completed the Foot Health Status Questionnaire. With the exception of age, absolute toe pressure and monofilament results, data was categorical in nature. Pearson’s Chi-square was used to identify significant relationships between variables.

**Results**

Thirteen percent of participants reported they were currently managing diabetes. This is below the 16.6% expected for Australians in the 65-74 years age range, however, the sample was a random representation of the local over-55 population. Of those reporting medical treatment for diabetes, significant findings suggest the feet of participants did not restrict work activities (p=0.01), climbing stairs (p=0.04), or the ability to shower and dress themselves (p=0.04). Significant interactions with a diagnosis of diabetes were also noted for an excellent self-rating of health (p<0.00) and energy level (p=0.04).

**Discussion**

The findings of this study indicate that the majority of older people living with diabetes are feeling healthy and are not restricted by their diagnosis. It is known that positive messaging in public health campaigns is more effective in changing behaviour than reinforcing negative information. Despite the need to educate clients about the risks associated with poorly controlled diabetes, clinicians also have a key role in highlighting the benefits of positive behavioural change and improving health literacy to enhance the health and wellbeing of clients.

### O32 Calf muscle stretching is ineffective in increasing ankle range of motion or reducing plantar pressures in people with diabetes and ankle equinus: a randomised controlled trial

#### Martin J Spink^1^, Angela Searle^1^, Christopher Oldmeadow^2^, Simon Chiu^2^, Vivienne H Chuter^1^

##### ^1^University of Newcastle, Ourimbah, NSW, Australia; ^2^Hunter Medical Research Institute, Newcastle, New South Wales, Australia

###### **Correspondence:** Martin J Spink

**Background**

Elevated plantar pressures have been implicated in the development and non-healing of foot ulcer in people with diabetes. Limited ankle dorsiflexion, or equinus, has been associated with elevated plantar pressures. This trial investigated if a stretching intervention could increase ankle dorsiflexion and reduce plantar pressures in people with diabetes.

**Methods**

Two-arm parallel randomised controlled trial at an Australian university podiatry clinic. Adults with diabetes and ankle equinus (≤ 5 degrees dorsiflexion) were randomly allocated to receive an eight-week calf stretching intervention (n=34) or continue with their normal activities for eight weeks (n=34). Primary outcomes were weight bearing and non-weight bearing ankle dorsiflexion range of motion and forefoot peak plantar pressures. Secondary outcome measures were forefoot pressure time integrals and adherence to the stretching intervention. Differences between groups were analysed with analysis of covariance.

**Results**

68 adults (mean (SD) age and diabetes duration 67.4 (10.9) years and 14.0 (10.8) years, 64.7% male) were recruited. Following the intervention, no significant differences were found between groups for ankle dorsiflexion either non-weight bearing (adjusted mean difference +1.3 degrees, 95% CI:-0.3 to 2.9, p=0.101) or weight bearing (adjusted mean difference +0.5 degrees, 95% CI:-2.6 to 3.6, p=0.743). No differences were found for forefoot in-shoe (adjusted mean difference 1.5kPa, 95% CI -10.0 to 12.9, p=0.803) or barefoot peak pressures (adjusted mean difference -19.1kPa, 95% CI:-96.4 to 58.1, p=0.628). Additionally no significant reductions in forefoot pressure time integrals were identified. Seven (20.6%) of the intervention group and 2 (5.9%) of the control group were lost to follow up.

**Discussion and Clinical Relevance**

Our data failed to show a statistically or clinically significant meaningful effect of calf muscle stretching on ankle range of motion, or plantar pressures, in people with diabetes and ankle equinus. Diabetes related changes to muscle-tendon or neural structures may make stretching less effective in this group. While current guidelines, including those from the American Diabetes Association, recommend stretching to maintain joint range of motion in people with diabetes, it is not effective as a stand-alone therapy to increase ankle joint range of motion in this population.

### O33 Diabetes education retention: A Systematic Review

#### Julia Yuncken, Cylie Williams, Terry Haines

##### Monash Univeristy, Melbourne, Vic, Australia

###### **Correspondence:** Julia Yuncken

**Background**

Diabetes education remains crucial for the treatment and prevention of diabetes complications. There is limited knowledge of long-term information retention or what are resultant behaviour changes due to diabetes education. There is also limited guidance for the most effective education methods to affect behaviour change. This systematic review evaluated the findings of the literary body on the impact that education practices have on behaviour change, knowledge or participant satisfaction.

**Methods**

Databases Medline, CINHAL, Science Direct, EMBASE, Web of Science and Cochrane were searched for articles investigating behaviour change, knowledge, or participant satisfaction in connection with diabetes education. The two reviewers screened articles independently against inclusion criteria and educational methods, and outcomes and timeframes were extracted from qualifying papers. Data were synthesized against Kirkpatrick’s Hierarchy of Learning.

**Discussion and clinical relevance**

In total, 849 studies were found using primary search criteria, of those all but 39 studies were disqualified using inclusion criteria. Of the 39 qualifying studies, 18 were randomised trials, nine were cross sectional studies and the remaining studies included quasi-experimental, observational and repeated measures design studies. Method of education included verbal, written and visual modes, delivered by both multi-disciplinary teams and single health care practitioners. Topics of education included general diabetes complications, foot complications, diet, physical activity and self-foot care. Outcomes reviewed included diabetes knowledge, foot care knowledge and HbA1C levels post education.

**Conclusion**

Diabetes education is ubiquitous in diabetes treatment however it remains unclear if patients retain the educational information provided or if the information retained causes behavioural change which in turn results in a decrease to complications.

### O34 Affective and cognitive factors associated with pain and function in people with plantar heel pain

#### Matthew P Cotchett^1^, Karl B Landorf^1^, Shannon E Munteanu^1^, Daniel Bonanno^1^, Glen Whittaker^1^, Angus Lennecke^1^, Virginia Medica^2^

##### ^1^Discipline of Podiatry, La Trobe University, Bundoora, VIC, Australia; ^2^Podiatry Department, The Northern Hospital, Epping, VIC, Australia

###### **Correspondence:** Matthew P Cotchett

**Background**

It is widely accepted that psychological variables, including affective, cognitive and behavioural factors, are associated with self-reported pain and self-reported physical function in patients with musculoskeletal pain. However, the relationship between psychological variables and foot pain and foot function, in people with plantar heel pain (PHP), has received less attention. Therefore, the aim of this presentation was to present the results from a series of cross sectional studies that evaluated a range of psychological variables in people with PHP.

**Methods**

Three cross sectional studies were conducted separately at different time points. The first cross sectional study evaluated 84 people with PHP to determine the association between depression, anxiety and stress with foot pain and foot function. This was followed by a second study involving 45 people with PHP and 45 people without PHP to determine if symptoms of depression, anxiety or stress increased the likelihood of having PHP. A third cross sectional study evaluated the association between kinesiophobia and catastrophising with foot pain and foot function in 36 people with PHP. Hierarchical and logistic regression models were used to evaluate the data.

**Results**

For participants with PHP, stress and depression scores were significantly associated with foot function but not foot pain. When the data was stratified by sex, stress and depression were significant predictors of foot pain and function in females. Symptoms of depression, anxiety and stress were found to increase the likelihood of having PHP. Finally, kinesiophobia and catastrophising were significantly associated with foot function, while catastrophising was associated with ‘first step’ pain in people with plantar heel pain.

**Discussion and clinical relevance**

In addition to addressing biological factors in the management of PHP, clinicians should consider the potential role of affective factors such as mood, and cognitive factors such as catastrophising and kinesiophobia which are equally important in the experience of pain in people with PHP. Podiatrists should contemplate the use of outcome measures to evaluate mood, anxiety, catastrophic thoughts and a fear of movement to help guide treatment that is viewed through a broader biopsychosocial model of health.

### O35 Insights from the Framingham Foot Study: Are key aspects of health being overlooked?

#### Marian Hannan

##### Marcus Institute for Aging Research, Hebrew SeniorLife and Harvard Medical School, Boston, MA, USA

Just as a good clinical examination asks directed queries about comorbidities and health to a patient to obtain an integrated understanding of the patient’s needs, so does a good research study add to our clinical insights. This session will use examples from the population-based Framingham Foot Study to highlight important key aspects of public health that may be overlooked yet essential perspectives as we consider the role that feet play in health and disease. Specific examples of current research evaluating the role of obesity will be presented. Important findings from the Foot Study will be discussed in relation to race/ethnicity, arthritis of the knee or hip, limitations in mobility, and disability. The need for early clinical intervention will be discussed as well as the evaluation of progression of foot conditions in persons prior to any clinical intervention. Finally, we will address what might be modifiable and what is not apt to change with clinical interventions.

Research findings may provide understanding of underlying mechanisms for the diseases we see in our patients. Epidemiology, as a specific focus, tells us the public health impact… and the impact of foot disorders is huge: in the general population, the risk of disability due to foot problems is much larger than heart problems, dementia or lung disorders. And despite public demand for understanding of foot health, many clinicians and public health advocates ignore the role of the foot.

Podiatry can merge with population sciences to encourage the integration of our knowledge so that key aspects of health are not overlooked. Our insights and public health knowledge are the keys to making a significant difference in foot health and advancing preventive efforts.

### O36 Preventing Overdiagnosis: how can we stop harming the healthy?

#### Ray Moynihan

##### Centre for Research in Evidence Based Practice , Bond University, Gold Coast, QLD, Australia

This presentation will offer an overview of the complex and counter-intuitive health challenge of overdiagnosis, which has been described as a ‘modern epidemic’ causing harm and challenging the sustainability of health systems. It will explore both the problem and potential solutions to it. The presentation will draw on national and international evidence and analysis, and demonstrate how Australia is at the forefront of national attempts to understand and address overdiagnosis.

In a nutshell, overdiagnosis happens when someone receives a diagnosis that does them more harm than good. It happens for example when a healthy person is diagnosed with a disease that will not actually ever cause them harm. The presentation will explain the nature of this vexing problem, and evidence which has attempted to estimate its extent across several conditions, including for example, thyroid, breast and prostate cancers.

The presentation will also explore what might be driving this problem, such as changes in diagnostic technology – often used in screening programs – which can identify ever-smaller abnormalities, many of which will highly likely never to go on to cause harm. Drawing on recent work published in the British Medical Journal, BMJ, the presentation will also explore a wide range of potential solutions.

A former award-winning journalist and author, Dr Moynihan is recognised internationally for his academic work on overdiagnosis, and the presentation will give a clear and compelling introduction to this problem and how we might collectively address it.

### O37 Overdiagnosis and overtreatment of musculoskeletal conditions in Australia and implications for podiatric practice

#### Rachelle Buchbinder

##### Monash Department of Clinical Epidemiology, Cabrini Institute, Department of Epidemiology and Preventive Medicine, School of Public Health and Preventive Medicine, Monash University, Melbourne, VIC, Australia

While early detection and treatment of disease brings benefits for many, there is increasing evidence of unnecessary testing and treatment, harming patients and diverting scarce resources from where they’re most needed. Overtesting and overtreatment is driven by many factors including clinician concern about missing diagnoses, increasingly sophisticated diagnostic tests which detect smaller and smaller abnormalities of uncertain prognosis, and screening programs that save lives but can also detect disease that won’t progress to cause harm. This leads to unnecessary treatment which can result in complications and harm to patients.

There are many examples of overtesting, overdiagnosis and overtreatment of musculoskeletal conditions. For example, an MRI of the knee will show some abnormality in 84% of people over the age of 50 years who have knee pain, and in 20% it will show meniscal damage. However these changes are equally as common in people without knee pain. Failing to take account of the prevalence of these abnormalities in the asymptomatic population can result in needless worry for the patient, and unnecessary treatments like arthroscopy. This cascade of unnecessary tests and treatments harms the patient and places a financial burden both on the individual and the health service.

This talk will provide examples of overdiagnosis and overtreatment relevant to podiatric practice in Australia and present some of the work of Wiser Healthcare, an Australian research collaboration that is investigating the cause and size of the problem and testing new solutions.

### O38 Palindromic rheumatism: Can arthritis that comes and goes hold the key to understanding RA?

#### Anne-Maree Keenan^1,2^, Kulveer Mankia^1,2^, Paul Emery^1,2^

##### ^1^Faculty of Medicine and Health, University of Leeds, Leeds, United Kingdom; ^2^NIHR Leeds Biomedical Research Centre , Leeds Teaching Hospitals Trust, Leeds, United Kingdom

###### **Correspondence:** Anne-Maree Keenan

Palindromic rheumatism (PR) a diagnostic label applied to episodes of self-limiting clinical arthritis, occasionally with skin disease. Symptoms are sudden onset of joint swelling which develops rapidly, lasts for a few hours or days then disappears. Attacks are usually mono-articular but with the site of joint pain often changing with each episode. While symptoms may affect any joint in the body with wrists, knees, shoulders, feet and ankles (in order of frequency) are most susceptible. Of note, almost half of patients with PR later develop rheumatoid arthritis (RA): understanding these patients may hold the key to understanding triggers for RA. Treatment for PR varies, but is generally centred on reducing inflammation and pain. The clinical appearance, differential diagnosis and imaging appearance is important for podiatrists to identify PR and refer on for early treatment

This presentation will also present initial findings from our Birmingham-Leeds collaboration. With colleagues at Birmingham, we have identified the substantial patient burden of PR: patients experience a wide range of physical symptoms and report psychological and emotional distress, frequently exacerbated by a lack of information and the apparent therapeutic and prognostic uncertainty. Early results from imaging studies at Leeds has indicated for the first time that active PR demonstrated greater peri-articular soft tissue inflammation compared to active synovitis that is seen with RA, potentially explaining the difference in clinical symptoms.

## Poster presentations

### P1 Are standard neuro-vascular assessments telling us more than we realised?

#### Emma Baker, Luke Donnan, Anna Horn, Caroline Robinson

##### Charles Sturt University, Thurgoona, NEW SOUTH WALES, Australia

**Background**

Neurovascular assessments are conducted routinely by podiatrists as a means of screening for peripheral arterial disease and peripheral neuropathy, to reduce the risk of foot ulceration, infection and amputation. Assessments commonly used to derive clinical data include the palpation of pulses, and the use of Doppler ultrasounds, systolic toe pressure sensors, monofilaments and tuning forks. Whilst it is common practice to use these assessments to monitor foot health, this data has the potential to illuminate a much more holistic perspective of a person’s health status.

**Methods**

Fifty participants (19 male, 31 female; 71.78±9.64 years) were recruited using random sampling to participate in a foot health promotion event at the Charles Sturt University Community Engagement and Wellness Centre. Basic neurovascular assessments were completed by student practitioners to determine each patient’s neurological, arterial and venous status. The Foot Health Status Questionnaire was also completed by all participants. Age, monofilament results and absolute toe pressures were recorded as continuous values, while all other data was categorical. Statistical interactions were identified using Pearson’s Chi-square.

**Results**

Those participants who reported ability to lift or carry bags of shopping, showed a statistically significant relationship with adequate vibration sensation (p=0.04), a palpable tibialis posterior pulse (p=0.04) and a triphasic Doppler assessment (p<0.00). The ability to get up from a sitting position (p<0.00) and independent showering and dressing (p=0.04), shared a significant association with a 10/10 monofilament score. It was also noted that a palpable tibialis posterior pulse shared a significant relationship with never having foot pain (p<0.00) aching feet (p=0.01), and no limitation in occupational capacity (p=0.02).

**Discussion**

These findings show significant interactions between standard neurovascular assessments and a range of activities of daily living. While the authors are mindful that these findings are correlational, not causational, a global view of neurovascular assessment may allow patient results to be viewed more holistically. Determining the ability to detect and heal tissue damage will not change, however, neurovascular findings may be used to stimulate further discussion about the patient as a whole, not just the presenting condition.

### P2 A dancer’s foot in turnout: A multi-segment kinematic study

#### Sarah L Carter^2,1^, Alan Bryant^2^, Luke S Hopper^1^

##### ^1^Western Australian Academy of Performing Arts, Edith Cowan University, Mount Lawley, WA, Australia; ^2^Podiatric Medicine and Surgery Division, School of Allied Health, University of Western Australia, Nedlands, WA, Australia

The full article version of this abstract has already been published and can be found at https://jfootankleres.biomedcentral.com/articles/10.1186/s13047-019-0318-1.

### P3 Idiopathic toe walking research update

#### Antoni Caserta^1,2^, Cylie Williams^2,3^, Prue Morgan^2^

##### ^1^Monash Health, Pakenham; ^2^Monash University, Frankston; ^3^Peninsula Health, Frankston

Idiopathic toe walking (ITW) is a diagnosis of exclusion for children who walk on their toes, with no medical cause. This presentation summarises two recent systematic reviews, a Cochrane review, and a systematic review of published outcome measures used for ITW.

The Cochrane review aimed to assess the effects of conservative and surgical interventions on gait normalisation, ankle range of motion, and pain in children with ITW, identifying adverse effects of the interventions and the frequency of recurrence. The systematic review aimed to identify and evaluate the clinical utility, validity and reliability of the outcome measures and tools used to quantify lower limb changes within this population.

Both reviews registered a protocol for search criteria, and data extraction.

There were 4 studies included within the Cochrane review, however only one study had data extracted reporting the difference between Botulinum Toxin A in addition to serial casting versus serial casting alone. These results indicated there was no clinically important differences

between the two intervention groups for improvement in toe walking to under 50% at 12 months as reported by parents, no change in passive ankle joint dorsiflexion range of movement, or no change in recurrence of toe walking gait at 12 months.

There were 27 articles included for data extraction within the systematic review. Results indicated that interventional studies were more likely to report range of motion and gait analysis outcomes, than observational studies. The Alvarez classification tool in conjunction with Vicon motion system, appeared as the contemporary choice for describing ITW gait. There were no significant associations between the use of range of motion and gait analysis outcomes and any other outcome tool or assessment in all studies. There were limited reliability and validity reporting for many outcome measures.

The results of the Cochrane review indicate a need for future good quality, interventional, multiple-armed randomised control trials for treatment of ITW and to include functional outcomes for treatment effectiveness measurement.The systematic review also highlighted that a consensus statement should be considered to guide clinicians and researchers in the choice of the most important outcome measures for this population.

### P4 The effectiveness of non-surgical intervention (foot orthoses) for paediatric flexible pes planus - a systematic review update

#### Sindhrani Dars, Helen Banwell, Hayley Uden, Saravana Kumar

##### University of South Australia, Adelaide, SA, Australia

The full article version of this abstract has already been published and can be found at https://journals.plos.org/plosone/article?id=10.1371%2Fjournal.pone.0193060.

### P5 The footwear experiences of people with gout a qualitative study

#### Mike Frecklington^1^, Anita Williams^2^, Nicola Dalbeth^3,4^, Peter McNair^1^, Peter Gow^5^, Keith Rome^1^

##### ^1^Health and Rehabilitation Research Institute, AUT University, Auckland, New Zealand; ^2^School of Health Science, University of Salford, Salford, United Kingdom; ^3^Department of Medicine, University of Auckland, Auckland, New Zealand; ^4^Auckland District Health Board, Auckland, New Zealand; ^5^Counties Manukau District Health Board, Auckland, New Zealand

The full article version of this abstract has already been published and can be found at https://jfootankleres.biomedcentral.com/articles/10.1186/s13047-019-0349-7.

### P6 Effects of a footwear intervention on foot pain and disability in people with gout a randomised controlled trial

#### Mike Frecklington^1^, Nicola Dalbeth^2,3^, Peter McNair^1^, Trish Morpeth^1^, Alain C Vandal^4,5^, Peter Gow6, Keith Rome^1^

##### ^1^Health and Rehabilitation Research Institute, AUT University, Auckland, New Zealand; ^2^Department of Medicine, University of Auckland, Auckland, New Zealand; ^3^Auckland District Health Board, Auckland, New Zealand; ^4^Department of Biostatistics & Epidemiology, AUT University, Auckland, New Zealand; ^5^Research & Evaluation Office, Ko Awatea, Counties Manukau Health, Auckland, New Zealand; ^6^Counties Manukau District Health Board, Auckland, New Zealand

The full article version of this abstract has already been published and can be found at https://arthritis-research.biomedcentral.com/articles/10.1186/s13075-019-1886-y.

### P7 Effects of new and worn footwear on plantar pressure in people with gout

#### Mike Frecklington^1^, Nicola Dalbeth^2,3^, Peter McNair^1^, Alain C Vandal^4,5^, Peter Gow^6^, Keith Rome^1^

##### ^1^Health and Rehabilitation Research Institute, AUT University, Auckland, New Zealand; ^2^Department of Medicine, University of Auckland, Auckland, New Zealand; ^3^Auckland District Health Board, Auckland, New Zealand; ^4^Department of Biostatistics & Epidemiology, AUT University, Auckland, New Zealand; ^5^Research & Evaluation Office, Ko Awatea, Counties Manukau Health, Auckland, New Zealand; ^6^Counties Manukau District Health Board, Auckland, New Zealand

**Background**

Footwear with good characteristics offers short-term improvements in foot pain and disability in people with gout, however, these are not sustained in the long-term. One reason for this may be wear and reduced structural integrity of the shoe over the six-month period. This study tested the effects of wear by comparing the plantar pressures in athletic shoes that had been worn for six months, compared to a new shoe of the same model and size in people with gout.

**Methods**

40 people with gout participated in a cross-sectional repeated measures study. Plantar pressure variables (peak plantar pressure and pressure time integrals) across seven regions of the foot were measured in random order in two footwear conditions; (1) a new pair of the same model of footwear (new footwear) and; (2) a pair of commercially available athletic footwear that had been worn for six months (worn footwear). Data were analysed using linear mixed models.

**Results**

The worn shoes had higher medial midsole hardness (P<0.0001), lateral midsole hardness (P<0.0001) and heel midsole hardness (P<0.0001). Signs of outsole wear was evident in the worn shoes, with the majority displaying normal upper (P<0.0001), midsole (P=0.05) and outsole (P<0.0001) wear patterns. No significant differences in peak plantar pressures were observed across the seven masked regions (P<0.007). Lower pressure time integrals were observed at the first metatarsophalangeal joint (P<0.0001), second metatarsophalangeal joint (P<0.0001) and hallux (P=0.003) with the worn shoes compared to the new shoes, consistent with off-loading of this area.

**Discussion and clinical relevance**

Signs of upper, midsole and outsole wear were evident in the worn footwear following six-months of use. These changes in the mechanical properties of the footwear may impact foot function, as observed by pressure time integral reductions at at the first metatarsophalangeal joint, second metatarsophalangeal joint and hallux.

### P8 Utilising foot posture index (FPI) as a static assessment in orthotic dispense fitting for hypermobile pes planus

#### Marabelle Heng^1,2^, Melissa Phua^3^

##### ^1^Singapore General Hospital, Singapore, SINGAPORE; ^2^University of South Australia, Adelaide, Australia; ^3^Tan Tock Seng Hospital, Singapore

**Background** Structural deviations in the foot and ankle predispose patients to changes in load-bearing, muscle imbalances and dynamic gait, resulting in compensatory strategies and overuse injuries. Orthotics are often fitted in such cases. During an orthotic dispense fitting, a podiatrist checks the static and dynamic response of the patient’s foot to the orthotic. The orthotic calcaneal stance position (OCSP) is a static objective measure, however it only shows the calcaneus positional changes, and does not reflect posture changes to the talus, mid foot and forefoot. Internationally, there is no common standard objective measures for static assessment during orthotic dispense fitting.

The foot posture index (FPI) is a validated clinical assessment tool that considers the foot positions in all planes (frontal, transverse, and saggital) and various anatomical segments (forefoot, mid foot and rearfoot). In the authors’ clinics, foot posture index (FPI) is used as a static assessment in orthotic dispense fitting in reducible, correctable conditions such as hypermobile pes planus.

**Methods** FPI was taken (i) weightbearing barefooted (‘before’) and (ii) in orthotic stance (OS; ‘after’). We sampled a total of 20 hypermobile pes planus feet with orthotic intervention across the two clinics. Data was deidentified. Mean and standard deviation for each FPI component was computed and ‘before-after’ difference were tested using paired t-test.

**Results** Mean (SD) of barefoot FPI = 9.9 (1.5); orthotic stance FPIos = 3.9 (1.7). Mean difference in ‘before-after’ FPI = 6.0 (p <.001). **Discussion & Clinical Relevance** The orthotic stance FPI (FPIos) is able to assess and score foot posture corrections achieved through the orthotic prescription in hypermobile pes planus. Hypermobile flat feet (FPI≥6) were corrected to within normal range. The FPIos could be a way forward in improving documentation of clinical outcomes in podiatric setting. Future studies could assess treatment outcome in large populations to establish norms and expected outcomes. Established norms and expected outcomes could in turn increase patients’ confidence and manage clients’ expectations in podiatric orthotic intervention.

### P9 Management of hallux valgus by general practitioners in Australia

#### Hylton B Menz, Glen A Whittaker, Shannon E Munteanu, Karl B Landorf

##### School of Allied Health, La Trobe University, Bundoora, VIC, Australia

**Background**

Hallux valgus is a common and disabling foot condition, however very little is known about how frequently hallux valgus presents to general practitioners (GPs), the treatments they provide, or the extent to which they refer patients to other health professionals.

**Methods**

We analysed data from the Bettering the Evaluation and Care of Health Program April 2000 to March 2016 inclusive. Patient and GP encounter characteristics were extracted. Data were classified by the International Classification of Primary Care, Version 2 (ICPC-2) using two

codes: *hallux valgus* (ICPC-2 L98007) and *bunion* (ICPC-2 L98001). Data were summarised using descriptive statistics and 95% confidence intervals (95% CIs) around point estimates.

**Results**

The dataset included 1,568,100 patient encounter records among which hallux valgus was managed 658 times, which equates to an estimated 60,000 GP encounters annually. Females accounted for 82% of all hallux valgus encounters, and the management rate was highest among patients aged 45 to 64 years. Hallux valgus was most frequently managed by referral to orthopaedic surgeons (28 per 100 encounters), counselling or advice (25 per 100) and referral to podiatrists (16 per 100). Pharmacological management was not frequently used (20 per 100) but primarily involved prescription of non-steroidal anti-inflammatory drugs (4 per 100).

**Discussion and Clinical Relevance**

Hallux valgus is a commonly encountered problem in Australian general practice but is mostly managed by referral to orthopaedic surgeons or podiatrists. Further research is required to examine the factors that influence the selection of surgical and non-surgical treatment pathways by Australian GPs.

### P10 “You don’t talk about feet”: Understanding parent concerns about foot health in infancy and early childhood

#### Lisa Hodgson^1^, Charlotte Growcott^2^, Anita Williams^2^, Chris Nester^2^, Stewart C Morrison^1^

##### ^1^School of Health Sciences, University of Brighton, Eastbourne, East Sussex, United Kingdom; ^2^School of Health & Society, University of Salford, Salford, UK

**Background**

There are considerable changes to the structure and function of the feet during infancy and early childhood. These changes typically reflect the plasticity of the feet but can cause concerns for parents, and lead to additional burden on health services. The aim of this study was to explore parent’s knowledge, practice and perceptions of foot health in infancy and early childhood.

**Methods**

A qualitative design was adopted and two researchers conducted semi-structured, one-to-one interviews with parents of children aged five years and under. Two researchers undertook data collection and a flexible, time-efficient approach was adopted. Interviews were conducted face-to-face, via telephone or via online telecommunication applications (Sturges & Hanrahan, 2004). All transcripts were transcribed verbatim and thematic analysis was used to explore the data. Coding and theme development were undertaken inductively and directed by the content and responses within the data (Braun & Clarke, 2006). Informed consent was obtained from all participants and ethical approval granted by the host institution(s) prior to undertaking the work.

**Results**

Eighteen interviews were conducted. Ten parents had one child under the age of five-years and eight parents had more than one child within this age range. Seven themes were identified relating to: (1) what parents understand about foot health in childhood; (2) how parents use and share information about foot health; (3) activities for supporting good foot health and development; (4) footwear choices and beliefs; (5) the role of health professionals; (6) accessing and understanding information; (7) developing practices to support parents.

**Discussion and Clinical Relevance**

The findings from this study provide insight into how parents view foot health in early infancy and childhood. The study depicts how parents learn about their children’s feet, receive and access support for their concerns, and what parents believe to be important. The findings from this work highlight that parents want accurate, accessible foot health information and that health professionals have an important role in supporting parents with easily identifiable, trustworthy sources that convey consistent advice.

1. Sturges, JE, Hanrahan KJ. Comparing telephone and face-to-face qualitative interviewing: A research note. Qualitative Research 2004; 4:107-118.

2. Braun V, Clarke V. Using thematic analysis in psychology. Qualitative Research Psychology. 2006; 3:77–101.

### P11 Foot-care needs in children and young people with intellectual and developmental disabilities

#### Stewart C Morrison, Laura Barrett, David Haines

##### School of Health Sciences, University of Brighton, Eastbourne, East Sussex, United Kingdom

**Background**

Healthcare needs in children and young people with intellectual and developmental disabilities are high. Foot problems have been reported to be very common in adults with intellectual disabilities; foot problems in children and young people are however, poorly understood. The aim of this study was to explore foot-care needs of children and young people with intellectual and developmental disability.

**Methods**

An exploratory, cross-sectional online survey was undertaken across a four-month period. A purposive snowball sampling approach was adopted in which parents / caregivers of children and young people with a diagnosis of intellectual or developmental disability were recruited. The survey tool was piloted before launch and comprised three domains. The first domain elicited information relating to parent/caregiver status, diagnosis of intellectual or developmental disability, secondary diagnoses, and age of the child/young person. The second domain explored foot-care and sought to obtain information about foot problems, access to health services, and existing knowledge about foot health. The third domain focused on footwear. Prior to launching the survey, ethical approval was granted from the host institution and all participants provided electronic consent before completing the survey.

**Results**

Complete data was collected from 49 respondents. Forty-five were parents and four were caregivers of a child or young person with intellectual and/ or developmental disability. Eighteen of the children had a diagnosis of Down syndrome and seven had a rarer chromosomal deletion (e.g Potocki-Lupski syndrome). Seventeen reported developmental disability (e.g. autism). Foot problems were common (75%) and tended to be musculoskeletal. ‘Pes planus’ was the most common foot concern (51%). Twenty-four parents/caregivers reported difficulties with finding appropriate shoes (48%) and this was often due to a mismatch between foot shape and the shoe (30%), or due to complications with orthotic devices (14%).

**Discussion and Clinical Relevance**

The results from this survey demonstrated that the burden of foot problems in children and young people with intellectual and developmental disabilities is considerable. The data offers a snapshot of the concerns that parents/caregivers encounter and the findings highlight the importance of access to foot-care services for these children and young people.

### P12 The effect of dynamic movement tasks on the restrictive properties of rigid strapping tape

#### Ainslee M. O'Connell, Herbert F. Jelinek, Luke Donnan

##### Charles Sturt University, Wodonga, VICTORIA, Australia

**Background:**

Ankle injuries are one of the most common sporting injuries in athletes. The combination of plantarflexion and inversion during jumping and cutting tasks increases vulnerability to a lateral ankle sprain injury during dynamic sports. Taping modalities are routinely used to reduce the risk of ankle injury, even though some research suggests the restrictive properties of tape may reduce during exercise. The purpose of this study was to determine whether a time point exists where the restrictive properties of prophylactic ankle taping reduces.

**Method:**

Forty-two participants (29 men, 13 women; age: 22.09±2.79yrs; height: 180.78±8.45cm; mass: 79.35 ±13.35kg) were recruited to complete a repeat measures, randomized control trial. Participants with taped (n=21) or untaped ankles (n=21) completed sets of ten cut-tasks at zero minutes, and again at five-minute intervals up to and including 20 minutes. A dynamic fatigue protocol was also completed between sets of cut-task trials to simulate sporting activity. Measures of ankle angles at the selected temporal events, peaks of gastrocnemius, fibularis longus and vastus medialis muscle activity, heart rate (HR) and rating of perceived exertion (RPE) were collected.

**Results:**

Significant increases in RPE and HR were noted in both the taped and non-taped groups at all time points, suggesting fatigue effects were consistent across both groups. The taped group showed significant increases in foot adduction at initial contact from 0-10min (p=0.03), 0-15min (p=0.04) and 0-20min (p≤0.001), and increased peak ankle dorsiflexion from 0-15min (p≤0.001). Non-significant increases in plantar-flexion were also observed from 15-20 minutes. The taped-group showed a significant increase in gastrocnemius activation during the load acceptance phase from 10-20mins (p≤0.05). Sagittal and transverse plane ankle angle peaks were also shown to converge from 15-20 minutes, although the taped group consistently displayed a reduced range of motion.

**Discussion:**

The findings of this study indicated that the restrictive benefits of self-adherent strapping tape decreased following 15 minutes of fatigue inducing dynamic activity, although the available movement is still less than the non-taped group. An awareness of such functional limitations following dynamic movement tasks will enable more appropriate use of ankle taping as an injury prevention tool.

### P13 Muscle strength differences in people with and without plantar heel pain

#### John W Osborne, Hylton B Menz, Glen A Whittaker, Karl B Landorf

##### Podiatry, La Trobe University, Bundoora, Victoria

**Background:** Plantar heel pain is a common condition but little is known about the relationship between muscle strength and plantar heel pain.

**Objectives:** To review the evidence relating to muscle strength in those with and without plantar heel pain.

**Methods:** We systematically reviewed the literature by searching key databases. Included studies assessed muscle strength (or endurance or size as proxies) in those with and without plantar heel pain. A modified quality index was used to assess risk of bias. Meta-analysis was performed where possible.

**Results:** Seven studies met the eligibility criteria. The quality of studies was generally high (score range 11 to 16 out of 17). Hallux plantarflexion, lesser toe plantarflexion, ankle dorsiflexion, ankle inversion and ankle eversion strength were reduced in those with heel pain compared to those without, however there was inconsistency in the findings between studies. Calf muscle endurance found no difference between those with and without plantar heel pain. Generally, foot muscle volume was smaller for people with plantar heel pain compared to those without.

**Conclusion:** People with plantar heel pain have reduced strength and volume of the foot muscles, but there is no discernible change in calf muscle endurance. It is unclear whether muscle weakness is a cause or consequence of plantar heel pain. However, these findings suggest that the role of muscle strengthening in the treatment of plantar heel pain is worthy of further investigation.

